# Targeted Delivery of Iron Oxide Nanoparticle-Loaded Human Embryonic Stem Cell-Derived Spherical Neural Masses for Treating Intracerebral Hemorrhage

**DOI:** 10.3390/ijms21103658

**Published:** 2020-05-22

**Authors:** Min Kyoung Kang, Tae Jung Kim, Young-Ju Kim, Lamie Kang, Jonghoon Kim, Nohyun Lee, Taeghwan Hyeon, Mi-sun Lim, Hee Jung Mo, Jung Hwan Shin, Sang-Bae Ko, Byung-Woo Yoon

**Affiliations:** 1 Department of Neurology, Seoul National University Hospital, Seoul 03080, Korea; eiri616@hanmail.net (M.K.K.); ttae35@gmail.com (T.J.K.); neosjh2009@gmail.com (J.H.S.); sangbai1378@gmail.com (S.-B.K.); 2 Department of Neurology, Seoul National University College of Medicine, Seoul 03080, Korea; 3 Biomedical Research Institute, Seoul National University Hospital, Seoul 03080, Korea; nr_lab@hanmail.net (Y.-J.K.); lamie02@hanafos.com (L.K.); 4 Center for Nanoparticle Research, Institute for Basic Science (IBS), Seoul 08826, Korea; hotoyt@snu.ac.kr (J.K.); thyeon@snu.ac.kr (T.H.); 5 School of Chemical and Biological Engineering, Institute of Chemical Processes, Seoul National University, Seoul 08826, Korea; 6 School of Advanced Materials Engineering, Kookmin University, Seoul 02707, Korea; nohyunlee@kookmin.ac.kr; 7 Research and Development Center, Jeil Pharmaceutical Co. Ltd., Yongin-si, Gyeonggi-do 17172, Korea; mabbak37@hanmail.net; 8 Institute of Reproductive Medicine and Population, Medical Research Center, Seoul National University, Seoul 08826, Korea; 9 Department of Neurology, Hallym University Dongtan Sacred Heart Hospital, Gyeonggi-do 14068, Korea; hjungmo@gmail.com

**Keywords:** iron oxide nanoparticle, human embryonic stem cell, spherical neural masses, magnet-embedded helmet, targeted delivery, intracerebral hemorrhage

## Abstract

This study evaluated the potential of iron oxide nanoparticle-loaded human embryonic stem cell (ESC)-derived spherical neural masses (SNMs) to improve the transportation of stem cells to the brain, ameliorate brain damage from intracerebral hemorrhage (ICH), and recover the functional status after ICH under an external magnetic field of a magnet attached to a helmet. At 24 h after induction of ICH, rats were randomly separated into three experimental groups: ICH with injection of phosphate-buffered saline (PBS group), ICH with intravenous injection of magnetosome-like ferrimagnetic iron oxide nanocubes (FION)-labeled SNMs (SNMs* group), and ICH with intravenous injection of FION-labeled SNMs followed by three days of external magnetic field exposure for targeted delivery by a magnet-embedded helmet (SNMs*+Helmet group). On day 3 after ICH induction, an increased Prussian blue-stained area and decreased swelling volume were observed in the SNMs*+Helmet group compared with that of the other groups. A significantly decreased recruitment of macrophages and neutrophils and a downregulation of pro-inflammatory cytokines followed by improved neurological function three days after ICH were observed in the SNMs*+Helmet group. Hemispheric atrophy at six weeks after ICH was significantly decreased in the SNMs*+Helmet group compared with that of the PBS group. In conclusion, we have developed a targeted delivery system using FION tagged to stem cells and a magnet-embedded helmet. The targeted delivery of SNMs might have the potential for developing novel therapeutic strategies for ICH.

## 1. Introduction

Spontaneous intracerebral hemorrhage (ICH) accounts for 10–20% of all strokes, but are disproportionately responsible for 40% of case fatality and 60% of functional dependency after stroke [1]. Unfortunately, there are no effective radical therapies for restoring independence and quality of life after ICH. There are only conservative treatments including the control of blood pressure, osmotic therapy for perihematomal edema, and life-saving surgical management and rehabilitation [2].

It has been postulated that stem cell administration could alleviate the primary and secondary effects of ICH via anti-inflammation, anti-apoptosis, promotion of neurogenesis and angiogenesis, and tissue repair or replacement [3,4,5]. However, specific guidelines on the types, cell counts, effective time window, and route for administration of stem cell therapy in ICH treatment are not well established. Therefore, careful consideration of strategies for effective stem cell therapy is needed.

Of the various cell types investigated, embryonic stem cells (ESCs), neural precursors (NPs), and neural stem cells (NSCs) have become attractive tools for treating brain injury. In addition to the trophic effect and immunomodulatory power of stem cells, they could replace damaged cells via regenerating neural networks and restoring neurovascular functions following stroke [3,6,7,8]. Spherical neural masses (SNMs), which are an intermediate generation of human ESC-derived pure masses before differentiation into NPs, have several advantages of a sustainable and highly efficient differentiation ability. The advantages of SNMs which have homogeneity, long-term passage, high efficiency differentiation, ease of manipulation, storing, thawing, and production in large-scale made them potential candidates to treat human acute ICH [9,10].

Intravenous administration of stem cells is a feasible and practical method. However, the efficacy of delivery is limited since only a small number of injected stem cells migrate to the target organ [11,12]. For augmenting the innate capability of stem cells to migrate to target organs, the use of magnetically labeled stem cells in an external magnetic field has been investigated in ischemic stroke [13]. However, a magnetically targeted system under an external magnet exposure has never been studied in stem cell therapy for ICH.

Here, we investigated the potential of magnetosome-like ferromagnetic iron oxide nanocubes (FIONs)-loaded human ESC-derived SNMs to improve the transportation of stem cells to the brain, ameliorate brain damage from ICH, and recover the functional status after ICH, under an external magnetic field by a magnet attached to a helmet.

## 2. Results

**Figure 1:**
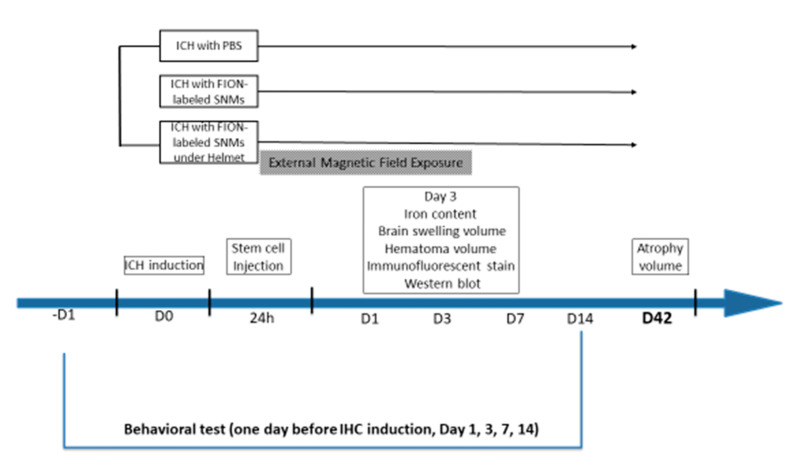
Schematic diagram representing the overall study design. Ferromagnetic iron oxide nanocubes (FIONs)–spherical neural masses (SNMs) were prepared from in vitro procedures before in vivo experiments. One day before the induction of intracerebral hemorrhage (ICH), rats were subjected to a behavioral test. On day 0, ICH was induced. On day 1, FION-labeled SNMs were injected. A magnet-embedded helmet was applied to the rats in the external magnetic field exposure group for three days after injection. On day 3, the rats were sacrificed for an analysis of the iron content, brain swelling volume, hematoma volume, immunofluorescent staining, and Western blotting. Rats were decapitated on day 42 after ICH to calculate the atrophy volume. Behavioral tests were performed on days -1, 1, 3, 7, and 14 after injection.

The experimental scheme is presented in Figure 1.

### 2.1. Determination of the Concentration of Iron Oxide Nanoparticles for Cell Labeling

**Figure 2:**
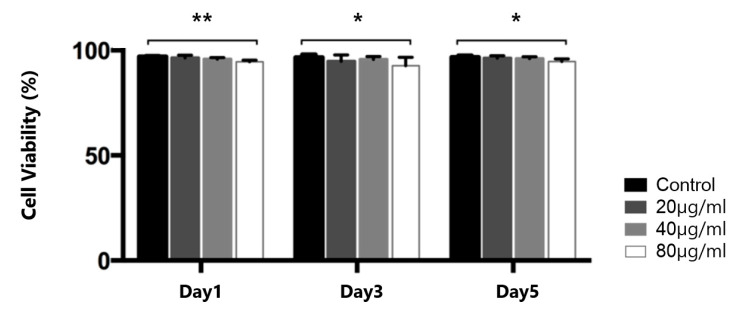
Cell viability assay of FIONs–labeled SNMs for 5 days. The relative cell viability is shown. (* *p* < 0.05, ** *p* < 0.005; *n* = 6 for each group).

We determined the toxic effect of the FIONs on the SNMs using the cell counting kit-8 (CCK-8) assay. To determine the optimal concentration to balance the effectiveness of the tagging and cellular toxicity, a cell viability analysis was performed at three different concentrations of FIONs (20, 40, and 80 µg/mL), as shown in Figure 2. There was no significant difference between the numbers of cells in the control vs. the presence of the 20 µg/mL and 40 µg/mL FIONs on days 1, 3, and 5 (day 1, 98.0 ± 2.3% and 96.9 ± 1.8%; day 3, 93.2 ± 5.5% and 95.2 ± 2.3%; day 5, 96.9 ± 5.2% and 95.7 ± 7.5%, respectively). There was a significant decrease in the viability of SNMs exposed to 80 µg/mL of FIONs (day 1, 93.1 ± 1.9%, *p* = 0.003; day 3, 90.2 ± 7.4%, *p* = 0.041; day 5, 93.2 ± 2.3%, *p* = 0.038, respectively). The concentration of 40 µg/mL FIONs was chosen based on the viability analysis.

### 2.2. Visualization of FIONs for Cell Labeling

**Figure 3:**
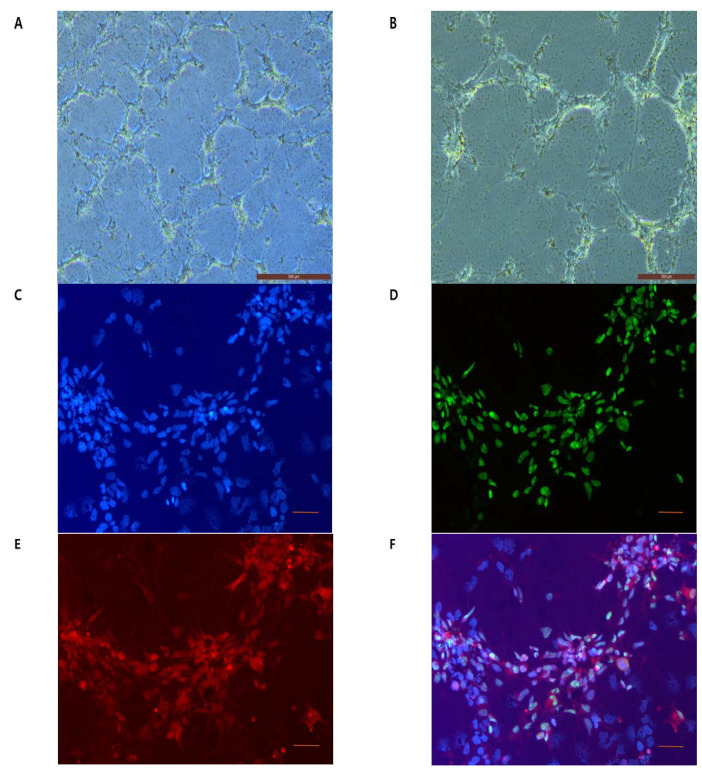
Morphological identification of FION-labeled SNMs in vitro. (**A**,**B**) Phase-contrast images of FION-labeled SNMs after 4 h of FION tagging. (**A**) Original magnification 5× (scale bars = 500 µm) (**B**) Original magnification 10× (Scale bars = 200 µm). (**C**–**F**) Fluorescence microscopy images of rhodamine B isothiocyanate (RITC)-conjugated-FION-labeled SNMs after 4 h of FION tagging. (**C**) 4,6-diamidino-2-phenylindole (DAPI) staining (blue), (**D**) human nuclear staining (green), (**E**) RITC staining (red), and (**F**) merged images were used to track FION-labeled SNMs. Original magnification 20× (scale bars = 50 µm).

To demonstrate the presence of SNMs labeled with FIONs, phase contrast optical imaging (Figure 3A,B) was performed for dissociated single SNMs with three-days incubation followed by a 4-h labeling with FIONs. The immunofluorescence imaging was performed on rhodamine B isothiocyanate (RITC)-conjugated FIONs (Figure 3C–F).

### 2.3. Measurement of Targeted Delivery of SNMs via Detection of FIONs by Prussian Blue Staining

**Figure 4:**
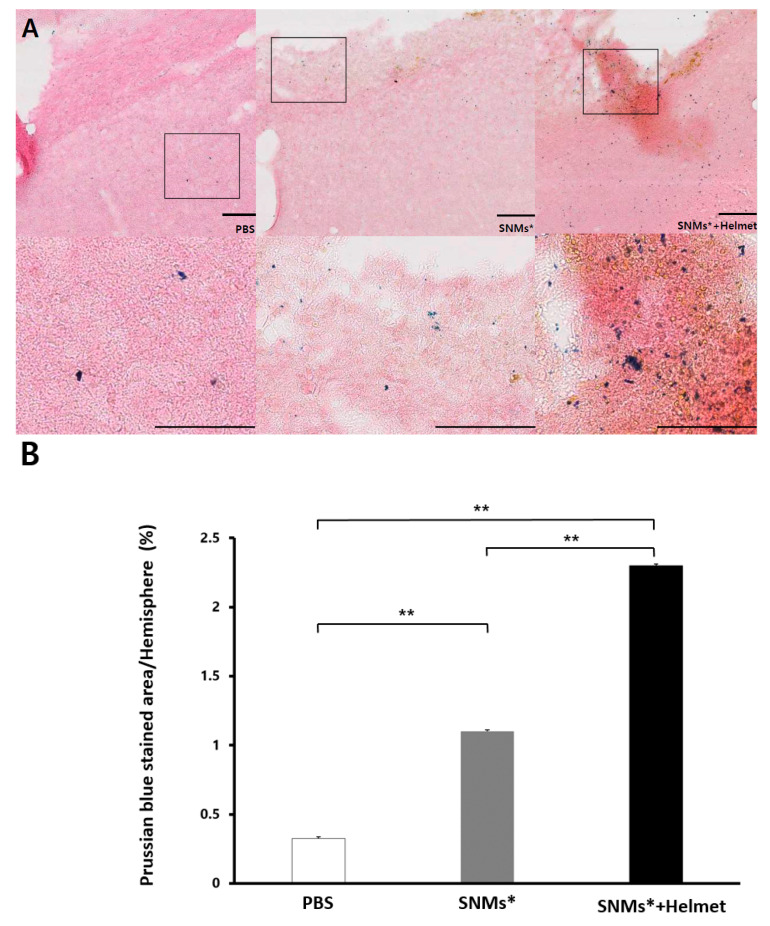
Prussian blue staining detects the presence of FION-labeled SNMs. (**A**) Prussian blue-stained ICH model with PBS, SNMs*, and SNMs*+Helmet groups. High-magnification images of the area outlined. Scale bar: 50 µm. (**B**) Relative percentage of Prussian blue-stained areas to hemispheric areas in the ICH model were analyzed using an image analyzer (* *p* < 0.05, ** *p* < 0.005; *n* = 3 for each group).

At day 3 post-ICH induction, optical imaging of the Prussian blue staining of the slides to identify FION-labeled SNMs was observed in the animals injected with phosphate-buffered saline (PBS group), FION-labeled SNMs (SNMs* group), and the group of FION-labeled SNMs under a magnet-embedded helmet application for three days (SNMs*+Helmet group, Figure 4A). The relative Prussian blue-stained area was significantly greater in the SNMs*+Helmet group than in the PBS or SNMs* groups (2.3 ± 0.02 mm^2^ vs. 0.3 ± 0.01 mm^2^, *p* < 0.001 and 1.1 ± 0.01 mm^2^, *p* < 0.001; respectively). There was a significant difference between the PBS and SNMs* groups (*p* < 0.001) (Figure 4B).

### 2.4. Targeted Delivery of SNMs Attenuated Swelling after ICH, but Not Hematoma

**Figure 5:**
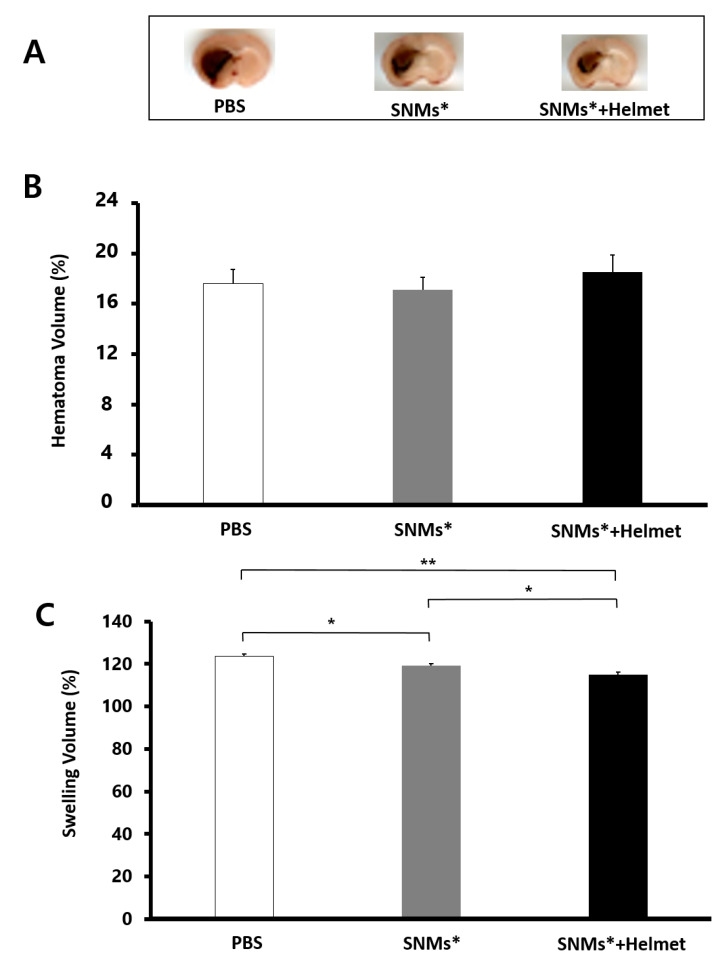
Measurements of the hematoma volume and swelling volume. (**A**) Photographic images of brain sections covering the perihematomal area in the ICH of the PBS, SNMs*, and SNMs*+Helmet groups. The absolute areas of hematoma and swelling in the ICH model were analyzed using an image analyzer (*n* = 6 for each group). (**B**) There was no difference among the hematoma volumes in the PBS, SNMs*, and SNMs*+Helmet groups. (**C**) The swelling volume decreased significantly in SNMs*+Hlemet group compared with that in the PBS and SNMs* groups. (* *p* < 0.05, ** *p* < 0.005).

Rats used in this experiment developed evident hemorrhages in the striatum (Figure 5A). The hematoma volume did not differ between the groups (PBS group, 17.1 ± 1.4%; SNMs* group, 16.4 ± 1.3 mm^3^; and SNMs*+Helmet group, 18.3 ± 1.6 mm^3^, as shown in Figure 5B), which indicated that the injection of SNMs* or helmet application did not affect the cerebral bleeding. Brain swelling at 72 h was attenuated in the SNMs*+Helmet group (115.1 ± 0.9%) compared with that in the PBS and SNMs* groups (123.7 ± 1.1%, *p* = 0.004; 119.1 ± 1.2%, *p* = 0.040, respectively, Figure 5C). The SNMs* group showed a significantly decreased swelling volume compared with that in the PBS group (*p* = 0.026).

### 2.5. Targeted Delivery of SNMs Reduced Recruitment of Inflammatory Cells

**Figure 6:**
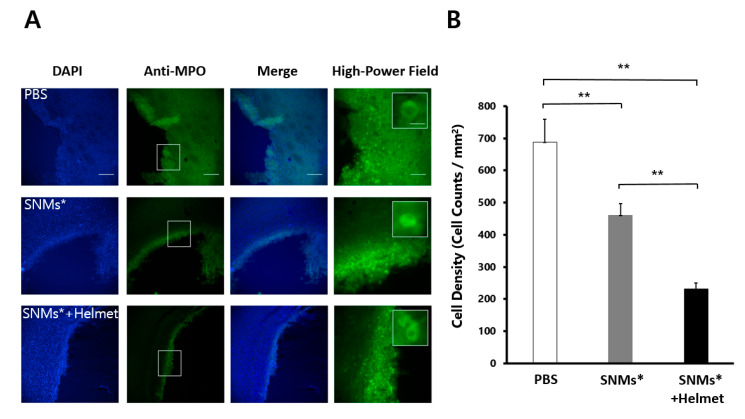
DAPI and anti-myeloperoxidase (MPO) in the perihematomal area after 3 days of ICH induction in the PBS, SNMs*, and SNMs*+Helmet groups. (**A**) Representative microphotographs are shown. High-magnification images of the area outlined. Scale bar of columns 1–3: 100 µm, column 4: 25 µm. (**B**) The density of the MPO-positive cells in the SNMs*+Helmet group was significantly lower than that in the PBS and SNMs* groups. (** *p* < 0.005; *n* = 3 for each group).

**Figure 7:**
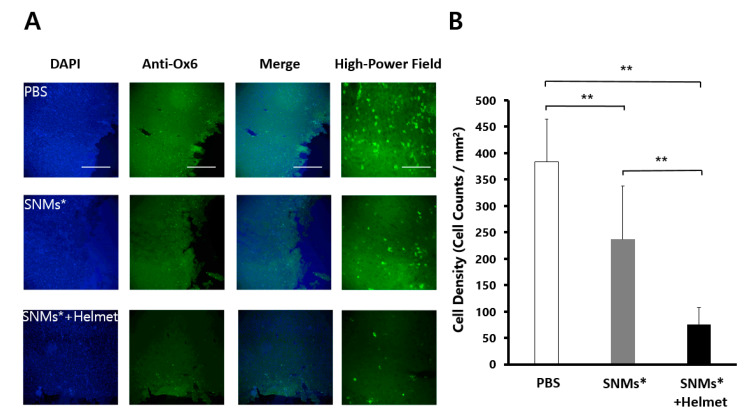
DAPI and monoclonal anti-Ox-6 antibodies in the perihematomal area after 3 days of ICH induction in the PBS, SNMs*, and SNMs*+Helmet groups. (**A**) Representative microphotographs are shown. High-magnification images of the area outlined. Scale bar of columns 1–3: 100 µm, column 4: 25 µm. (**B**) The density of the Ox-6-positive cells in the SNMs*+Helmet group was significantly lower than that in the PBS and SNMs* groups. (** *p* < 0.005; *n* = 3 for each group).

Three days after ICH induction, the anti-myeloperoxidase (MPO)-positive or anti-MHC Class II RT1B (Ox6)-positive cells clustered in a ring-like distribution around the perihematomal area (Figure 6A and Figure 7A). The quantitative analysis of immune cells showed a significant decrease in the density of MPO-positive cells (249 ± 18 cells/mm^2^) in the SNMs*+Helmet group compared with that in the PBS and SNMs* groups (690 ± 66 cells/mm^2^, *p* < 0.001; 474 ± 19 cells/mm^2^, *p* < 0.001, respectively, Figure 6B). In addition, the SNMs* group showed a significantly decreased MPO-positive cell density compared with that in the PBS group (*p* < 0.001). The same pattern was observed in the Ox-6-positive cell count, showing a significant decrease in the SNMs*+Helmet group (75 ± 44 cells/mm^2^) compared with that in the PBS and SNMs* groups (383 ± 79 cells/mm^2^, *p* < 0.001; 236 ± 110 cells/mm^2^, *p* < 0.001, respectively, Figure 7B). The FION-labeled SNMs* group showed a significantly decreased Ox-6-positive cell density compared with that in the PBS group (*p* = 0.001). These complementary results indicate that the injection of SNMs* and helmet application attenuated the immune response after ICH.

### 2.6. Targeted Delivery of SNMs Reduced COX-2 Mediated Inflammatory Response

**Figure 8:**
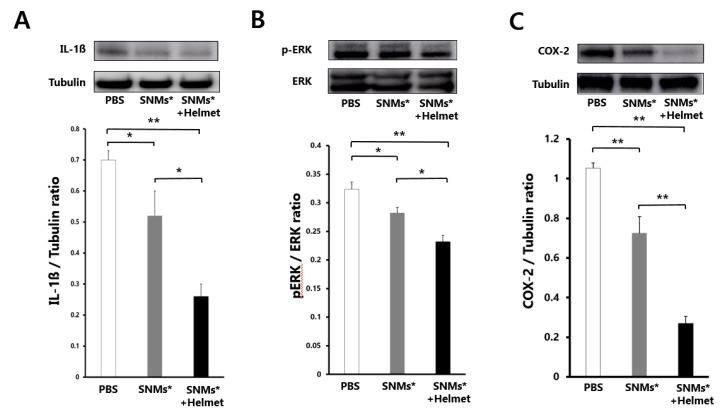
Western blot analysis after 3 days of ICH induction in the PBS, SNMs*, and SNMs*+Helmet groups. Photographic images and quantification analysis of (**A**) normalized IL-1ß, (**B**) proportion of extracellular signal-regulated kinase (ERK) and phosphorylated extracellular signal-regulated kinase (pERK), (**C**) normalized cyclooxygenase-2 (COX-2). (* *p* < 0.05, ** *p* < 0.005; *n* = 3 for each group in each test).

The SNMs* and SNMs*+Helmet groups were reduced in their levels of interleukin-1 beta (IL-1β), phosphorylated extracellular signal-regulated kinase (p-ERK), and expression of cyclooxygenase-2 (COX-2) in the perihematomal regions after three days of ICH induction. The relative optical density of IL-1β, compared with that of tubulin, decreased significantly in the SNMs*+Helmet group (0.26 ± 0.04) compared with that in the PBS and SNMs* groups (0.69 ± 0.08, *p* < 0.001; 0.52 ± 0.03, *p* = 0.008, respectively, Figure 8A). The SNMs* group showed a decreased IL-1β expression compared with that in the PBS group (*p* = 0.026). The normalized p-ERK ratio in the SNMs*+Helmet group (0.23 ± 0.01) also showed attenuated activation compared with the PBS (0.32 ± 0.01, *p* < 0.001) and SNMs* (0.28 ± 0.01, *p* = 0.036) groups. The SNMs* group showed the p-ERK expression with downregulation patterns compared with that in PBS group (*p* = 0.041) (Figure 8B). The normalized expression of the pro-inflammatory protein, COX-2, also decreased in the SNMs*+Helmet (0.20 ± 0.14, *p* < 0.001) and SNMs* groups (0.72 ± 0.10, *p* = 0.003) compared with that in the PBS group (1.05 ± 0.04). The effect of the application of a magnetic helmet was evident on the COX-2 expression (*p* < 0.001) (Figure 8C).

### 2.7. Measurement of Brain Atrophy Volume

**Figure 9:**
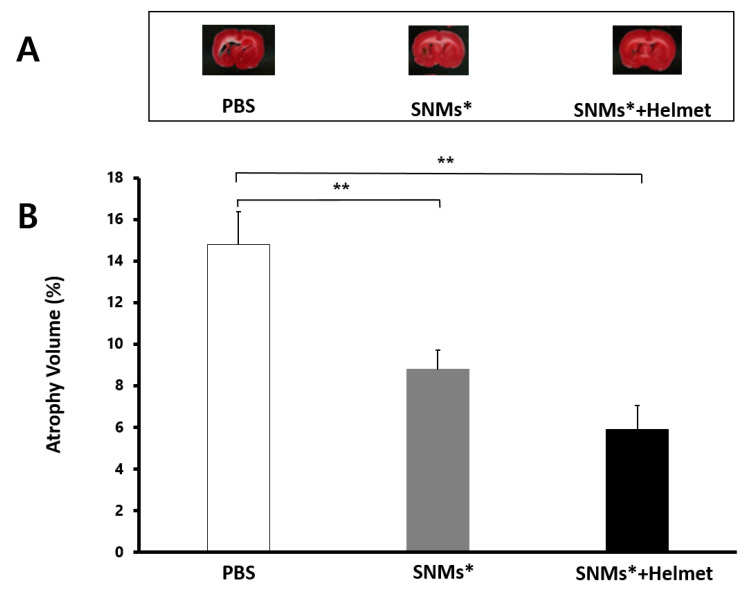
Atrophy volume after ICH induction. Atrophy size of rat brains was measured by triphenyl tetrazolium chloride-stained sections 42 days after induction of ICH. (**A**) Representative microphotographs are shown. (**B**) The atrophy volume in the ICH model, analyzed using an image analyzer, showed a significant decrease in the SNMs* and SNMs*+Helmet groups compared with that in the PBS group. (** *p* < 0.005; *n* = 6 for each group in each test).

The hemispheric area analysis conducted six weeks after ICH showed significantly decreased hemisphere atrophy in the SNMs*+Helmet and SNMs* groups compared with that in the PBS group (14.3 ± 1.58% vs. 8.8 ± 0.92%, *p* = 0.003; 5.9 ± 1.16%, *p* = 0.002, respectively, Figure 9). However, the SNMs*+Helmet group did not exhibit a significant difference compared with the SNMs* group (*p* = 0.132).

### 2.8. Targeted Delivery of SNMs Enhanced the Neurological Function Recovery in the Early Stage

**Figure 10:**
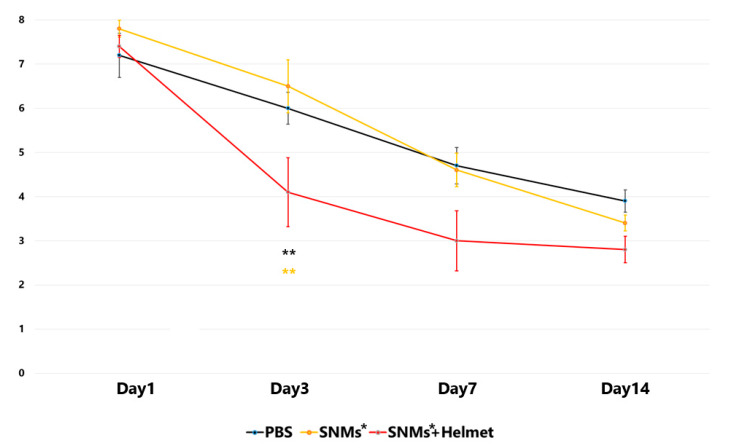
Behavioral functional tests were regularly performed for 2 weeks after the induction of ICH. The results of each group are represented by different color dots: black line, PBS group; yellow line, SNMs* group; red line, SNMs*+Helmet group. On day 3 after ICH, the rats in the SNMs*+Helmet group improved limb placement function compared with those in the PBS or SNMs* groups. (** *p* < 0.005; *n* = 6 for each group).

We evaluated neurological function from the day before to the 14th day after ICH induction using a limb placement test. The limb placement tests conducted the day before ICH scored 0 for all subjects in three groups. In the PBS group, the limb placement test score peaked (7.2 ± 0.5) one day after ICH and decreased to 6.0 ± 0.4 on day 3, 4.7 ± 0.4 on day 7, and 3.9 ± 0.2 on day 14. In the SNMs* group, the score was 7.8 ± 0.2 on day 1, 6.5 ± 0.6 on day 3, 4.6 ± 0.4 on day 7, and 3.4 ± 0.2 on day 14. In the SNMs*+Helmet group, there was a significant decrease on day 3 (7.4 ± 0.3 on day 1, 4.1 ± 0.8 on day 3, 3.0 ± 0.7 on day 7, and 2.8 ± 0.3 on day 14) compared with the PBS and SNMs* groups, with *p* < 0.001 and *p* = 0.002, respectively. (Figure 10) However, no significant difference was observed between the groups on days 1, 7, and 14 after induction of ICH. These results suggest that the targeted delivery of SNMs* improved neurological function recovery during the early phase of the disease.

## 3. Discussion

Our present study indicates that the targeted delivery of SNMs attenuated inflammatory cascades which resulted in secondary insults of ICH, and improved the neurological status during the early phase.

It is well known that there is no definite treatment for ICH. Approximately half of hemorrhagic strokes result in death within a month, wherein primary ICH and resultant brain edema are the key players [14,15]. There is increasing evidence that the inflammatory response around primary ICH contributes to the formation of edema, which exacerbates mass effect, augments the cell death through secondary ischemia, and causes further inflammatory insults to the surrounding brain tissue in turn [16,17]. In this study, a decreased activation of microglia or the recruitment of MPO-positive or Ox-6-positive inflammatory cells, and the downregulation of the IL-1β-ERK-COX-2 pathway in the inflammatory cells attenuated edema followed by no change in the primary hemorrhage volume, but decreased atrophy in the SNMs*+Helmet group. As other types of stem cells, mesenchymal stem cells were also proven to alleviate airway inflammation through the ERK-COX2 pathway, which was thought to be the mechanism of action of beneficial effects for ICH in this study [18]. Thus, the targeted delivery of SNMs might have the potential for emerging therapeutic strategies to prevent aggravation after primary ICH insults.

Due to the low migration and retention ability of stem cell transplantation, a magnetic nanoparticle was evaluated in various diseases including ischemic stroke, ischemic heart disease, and ischemic peripheral disease for the effects of tagging, homing, and retention which had proven by the immunological staining for differentiation, calcium repositioning staining, and secretion profiles for various growth factors and cytokines [13,19,20,21]. In several magnetic nanoparticles, we chose FIONs because of their high magnetization, safe pharmacological profile, ability to retain within cells, and incorporation into the iron store of the body in case of degradation [17,22]. Recently, the application of an external magnetic field was introduced in order to maximize the homing effects of stem cell with a magnetic nanoparticle [13,23]. However, a magnet on the direct surface of the skull or the wound, which was applied for the external magnetic field in the previous studies, is an invasive method for the treatment of humans. A magnet-embedded helmet is a feasible method for the application to humans.

The improvement in motor function evaluated by the limb placement test seen on day 3 was not observed on days 7 and 14, which may imply an effect during the early phase (within 72 h) of stem cell implantation in ICH. In general, edema formation and cellular toxicity are known to be maximum at day 3 after ICH [24]. The attenuation of edema followed by a decrease in inflammatory markers correlated with the improvement in neurological function on day 3. According to the INTERACT-II (Intensive Blood Pressure Reduction in Acute Cerebral Hemorrhage Trials) and VISTA (Virtual International Stroke Trials Archive) databases, most neurological deteriorations occur within 72 h and have a major effect on poor prognosis after stroke. Therefore, we decided to observe the protective effect of SNMs via an anti-cellular toxicity mechanism for three days after ICH [24,25]. However, there have been studies on delayed neurological deterioration from 72 h to 7 days after ICH. The mRNA expression of pro-inflammatory mediators was upregulated at least seven days after ICH [26,27]. Therefore, there was room for an extended inflammatory response after 72 h. In this xenograft-based short-term study which was mainly focused on the acute phase treatment of ICH, the effect of stem cell therapy was proven only on day 3. However, SNMs could have shown higher potential than that in the study because they could replace diseased cells in an in vivo setting through the subacute to chronic phase of ICH. We did not evaluate the differentiation of administered SNMs in this study due to the limitation of preclinical xenograft-based properties. Further research for the long-term application of a helmet after injection of SNMs, monitoring differentiation and transplantation in an in vivo environment, and a follow-up evaluation of the neurologic status in the subacute and chronic status is needed.

SNMs derived from human ESCs have several advantages. They can dissociate into a single cell suspension, proliferate more than 100-fold in total cell numbers, reproduce in neural restricted lineage, replace a damaged neurovascular unit, set in the appropriate anatomical sites in the brain, and suppress teratoma formation [9,10,28]. Compared with neurospheres which are sensitive to environments, have heterogeneity, a diminishing proliferation capacity after an extended number of passages, and low efficiency of differentiation, SNMs have several advantages of homogeneity, long-term passage, high efficiency differentiation, ease of manipulation, storing, thawing, and production in large-scale [10,29]. These properties made SNMs potential candidates to treat human acute ICH. SNMs were used in this study because it could be readily administered to the patients with ICH in the acute phase by thawing after large-scale production. In addition, by choosing SNMs themselves instead of neurons or NPCs derived from SNMs, we could avoid the possibility of heterogeneity and contamination accompanying further differentiations. However, SNMs could be more emboligenic because of their larger size compared with other types of stem cells. An evaluation of embolism of other organs is needed in the future.

It would be pathbreaking to develop an efficient delivery system of an intravenous injection of stem cells for the application of treating human ICH. There have been several studies on the intravenous injection of mesenchymal stem cells labeled with superparamagnetic iron oxide nanoparticles (SPIONs) under external magnetic fields [30]. FION, which is a smaller, faster, and more powerful magnetized tagging material compared with SPION, could be a more efficient targeted delivery material for stem cell transplantation [17,31,32]. This was the first study to use SNMs and FIONs under external magnetic fields for a targeted stem cell delivery system that combines the most feasible three players.

This study had some limitations. We could not check whether the stem cell transplantation and differentiation were efficiently performed in vivo in the rat model. Second, we could not explore the impact of FIONs on the therapeutic potential of SNMs compared with the naïve SNMs control or the effect of the magnet-embedded helmet itself on the acute phase of ICH. Third, an extensive behavioral test beyond motor function, long-term studies for differentiation in an in vivo environment, and explorative studies on other organs for embolism are needed. Furthermore, we could not determine the optimal timing for stem cell transplantation or the application of a helmet. In spite of these limitations, the concepts and results of the targeted stem cell delivery system in this study would shed light on translational stroke research.

## 4. Materials and Methods 

### 4.1. Differentiation of SNMs from Human ESCs

This study was approved by the institutional review board of the Seoul National University Hospital (IACUC No. 17-0075-C1A0). The SNMs were produced from the undifferentiated human ESC 43 line as described previously [9,14,15]. In brief, the pieces of undifferentiated SNU human ESC 43-line colonies were mechanically dissociated and cultured in a dish for 7 days to form embryoid bodies (EBs). The cultured EBs were attached to Matrigel-coated culture dishes and cultured to select neural structures consisting of NPs in media supplemented with 0.5% N2 for 5 days. The selected NPs were expanded to form rosette structures in an expansion media with basic fibroblast growth factor (Invitrogen, Carlsbad, CA, USA) and 1% N2 for another 4 days. These homogenous SNMs were purified and frozen. For labeling, SNMs were dissected every single SNM, as previously reported [16].

### 4.2. Iron Oxide Nanoparticles for Cell Labeling

FIONs were synthesized as described previously [17]. The size of the FIONs used was 57.8 ± 9.9 nm. Single SNMs were incubated 3 days before labeling with FIONs. The 4-h incubation was required to label the cells with FIONs as described previously [17]. SNMs were treated with various concentrations (20, 40, and 80 µg/mL) of FIONs. The cytotoxicity of FIONs was evaluated using a CCK-8 assay (Enzo Life Sciences Inc., NY, USA) for 5 days (*n* = 6 for each group). After adding 10 µL of CCK-8 solution per well, plates were incubated at 37 °C for an additional 3 h. The absorbance was assayed at 450 nm using a Plate Reader (VersaMax™, Molecular Devices, San Jose, CA, USA). Relative cell viability was calculated as previously described. [33]

(1) $$\mathrm{Relative}\;\mathrm{Cell}\;\mathrm{Viability}\;\left(\%\right)=100\times \frac{A_{FION}}{A_{Control}}$$

*A_FION_* and *A_Control_* were defined as the absorbance of the cells treated within various concentrations of FIONs and the control medium, respectively. SNMs incubated in the control medium were considered to be 100% viable compared with SNMs with FION-labeling (SNMs*). The FION content for injection was determined to be 40 µg/mL, based on the viability analysis.

To track SNMs labeling with FIONs, phase-contrast microscopy (ECLIPSE Ti, Nikon, Tokyo, Japan), preparation of RITC tagged FIONs, and immunofluorescent staining of cells were performed [33,34]. In brief, RITC was added to covalently bind 10 mg of FIONs in ethanol. After 8 h, the solution containing RITC-FION was centrifuged, washed with ethanol, and purified. The homogeneously purified single SNMs were incubated 3 days before labeling with RITC-FIONs, followed by 24-h incubation with RITC-FIONs. RITC-FION-SNMs were seeded on glass slides, followed by fixation in 4% formaldehyde. Each slide was washed with PBS (Sigma-Aldrich, St. Louis, MO, USA). The slides were incubated overnight at 4 °C with mouse monoclonal anti-human nuclei primary antibody to label the SNMs (1:100; Chemicon International, Temecula, CA, USA) as primary antibody, followed by incubation with the secondary antibody Alexa 488-conjugated goat anti-mouse IgG (1:200, Molecular Probes, Eugene, OR, USA) for 2 h at room temperature (22 ± 3 °C). Finally, the slides were counterstained with 4,6-diamidino-2-phenylindole (DAPI, Sigma, Taufkirchen, Germany) for cell nuclear staining. All slides were washed with PBS, and immunofluorescence was visualized and counted in the ipsilateral hemisphere using a laser scanning confocal microscope (ECLIPSE Ni-E, Nikon, Tokyo, Japan).

### 4.3. Induction of ICH, Intravenous Injection of FION-Labeled SNMs, and Application of a Magnet-Embedded Helmet for Targeted Delivery

**Figure 11:**
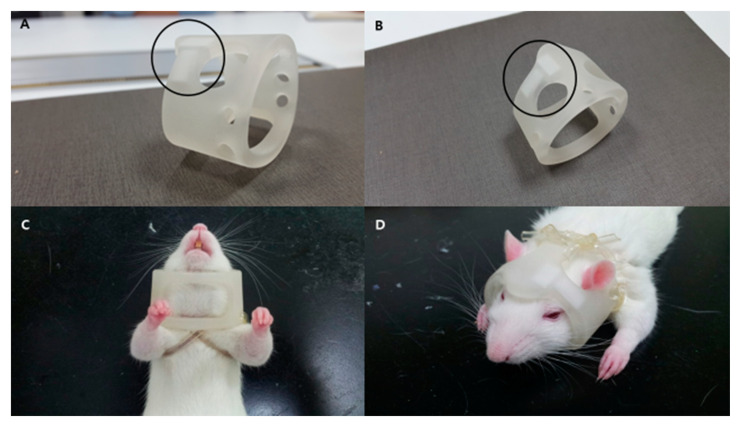
Photographs of the magnet-embedded helmet and its application. Two magnets were embedded under a helmet made by a 3D printing method, as shown in the (**A**) lateral and (**B**) oblique views. The black circles indicate the positions of the two magnets. The helmet was attached to the rat just to the extent that it would move freely with flexible polyvinyl chloride laboratory tubes, as shown in the (**C**) bottom and (**D**) top views.

A total of 108 male outbred Sprague-Dawley rats (Koatech, Seoul, Republic of Korea), 8–9-weeks-old and weighing between 230 and 330 g, were used in these experiments. We injected 0.4 unit of collagenase type IV (0.5 U in 1 µL PBS; Sigma-Aldrich, St. Louis, MO, USA) intrastriatally (3.0 mm left, 0.2 mm posterior to the bregma at 6.0 mm depth) using a stereotaxic method described previously [35]. At 24 h after ICH induction, the rats were randomly separated into three experimental groups: PBS group (500 µL), SNMs* group (4 × 10^6^ cells/500 µL), and SNMs*+Helmet group (4 × 10^6^ cells/500 µL) induced by two pieces of neodymium magnet (5 × 10 × 2 mm^3^, 0.32 T) embedded within a 3D-printed helmet (printer: ProJet 3500, 3D Systems Co., Littleton, CO, USA; plastic: SOFT Poly Lactic Acid-Flex, ORBI-TECH, Leichlingen, Germany). Two magnets were placed below the central and left side of the helmet, as shown in Figure 11 (patent number: KR/10-2017-0092408). A helmet with two embedded magnets (5 × 10 × 2 mm^3^, 0.32 T) was made by 3D printing methods (ProJet, CEP TECH, 2017, Korea). 

### 4.4. Measurement of Iron Content, Hematoma Volume, and Swelling Volume

At 3 days post-ICH induction, the rats were sacrificed for the measurement of the iron content, brain swelling volume, hematoma volume, and water content. After cardiac perfusion-fixation with 4% paraformaldehyde (PFA) in 0.1 mol/L PBS, the brains were removed and cut into 40-μm-thick coronal sections using cryostat to measure the iron content (Leica CM 1900, Leica Biosystems Nussloch GmbH, Nussloch, Germany) [35]. Prussian blue stain was used to identify FION-labeled SNMs (*n* = 3 for each group). The Prussian blue-stained area of the lesions was measured by image analysis and presented as the percentage of the blue-positive area in the entire hemisphere. (Image J, version 1.51c, National Institutes of Health, Bethesda, MD, USA). [13,36] Next, a 1-mm-thick brain section was fixed in 4% PFA for 24 h before the measurements of the hematoma and swelling volumes (*n* = 6 for each group) [37]. Briefly, the hematoma volume was defined as the summation of the clot area of each section (absolute hematoma volume) divided by the summation of the ipsilateral hemispheric area of each section, measured by image analysis (Image J, version 1.51c, National Institutes of Health, Bethesda, MD, USA) [38,39]. The dimensional analysis for the swelling volume was applied via image analysis, and defined as shown below (Image J, version 1.51c, National Institutes of Health, Bethesda, MD, USA) [40].

(2) $$\mathrm{Hematoma}\;\mathrm{Volume}\;\left(\%\right)=100*\;\frac{absolute\;hematoma\;volume}{ipsilateral\;hemispheric\;volume}$$

(3) $$\mathrm{Swelling}\;\mathrm{Volume}\;\left(\%\right)=100*hemispheric\;volume\;of\;\frac{ipsilateral}{contralateral}\;side$$

### 4.5. Immunofluorescence Staining and Cell Quantification

At 3 days post-ICH induction, immunofluorescent staining of brain tissues was performed using cryopreserved 40-μm-thick coronal sections after cardiac perfusion-fixation with 4% PFA in 0.1 mol/L PBS (*n* = 6 for each group) [41]. Briefly, each section was incubated with 0.5% bovine serum albumin (BSA) and 0.3% Triton X solution followed by 10% normal serum in PBS for 1 h. Sections were incubated with monoclonal anti-MPO antibodies (DAKO, Carpinteria, CA, USA) for activated neutrophil, and monoclonal anti-Ox-6 antibodies (Santa Cruz Biotech, Santa Cruz, CA, USA) for activated microglia/macrophages at 4°C for 16 h. After washing, each section was subsequently incubated for 2 h at room temperature with Alexa 488-conjugated goat anti-mouse antibodies (1:200, Molecular Probes, Eugene, OR, USA) as a secondary antibody. Cell nuclei were stained with DAPI (Sigma, Taufkirchen, Germany). Stained cells were examined under a confocal laser scanning biological microscope (ECLIPSE Ni-E, Nikon, Tokyo, Japan). To perform the quantitative analysis of the positively stained cells, two independent investigators (MK Kang and JH Shin), who were masked to the group allocations, examined the perihematomal regions in each section. Total counts in all measured sections were converted into cell densities [41].

### 4.6. Western Blot Analysis

At 3 days post-ICH induction, the rats were sacrificed and the extracted brains were frozen in liquid nitrogen for Western blot analysis followed by centrifugation of homogenates (*n* = 9 for each group) [41]. Next, 50 μg of protein was separated on a 1-D polyacrylamide electrophoresis gel (Mini-PROTEAN TGXTM, Bio-Rad, Hercules, CA, USA) and transferred to nitrocellulose membranes (Trans-Blot TurboTM, Bio-Rad, Hercules, CA, USA). The membranes were incubated in 5% BSA blocking buffer for 90 min, and the blots were tagged with antibodies against IL-1β (Abcam, Cambridge, UK), ERK, and p-ERK, (Cell Signaling Technology, Danvers, MA, USA), and COX-2 (Abcam, Cambridge, UK). Anti-tubulin antibody (Life Technologies Co., Carlsbad, CA, USA) was used for internal control. Immunoreactivity was examined using enhanced chemiluminescence (Millipore, Bedford, MA, USA), and the relative optical densities were determined by image analysis (Image J, version 1.51c, National Institutes of Health, Bethesda, MD, USA).

### 4.7. Measurement of Brain Atrophy Volume

At 42 days post-ICH induction, the rats were sacrificed and 1-mm-thick fresh brain slices were stained with triphenyl tetrazolium chloride for the measurement of hemispheric atrophy (*n* = 6 for each group). Total hemispheric brain volume was calculated by the summation of the hemispheric area in each section multiplied by the distance between sections via image analysis on digital photographs of the slices (Image J, version 1.51c, National Institutes of Health, Bethesda, MD, USA). Poststroke hemispheric atrophy volume was defined as described previously [42].

(4) $$\mathrm{Atrophy}\;\mathrm{Volume}\;\left(\%\right)=100*\left(1-hemispheric\;volume\;of\;\frac{ipsilateral}{contralateral}\;side\right)$$

### 4.8. Behavioral Testing

To determine whether the FION-labeled SNMs improved the motor deficit after ICH, behavioral tests were performed using a limb placement test on the day before induction of ICH, and on days 1, 3, 7, and 14 post-ICH induction (*n* = 6 for each group). The independent two raters (MK Kang and HJ Mo), who were masked to the group allocations, scaled the test. In the limb placement test, the rats were graded from 0 to 2 in each limb, as previously described: 0 points (placed limb normally), 1 point (placed limb with a delay of more than 2 s and/or incompletely), and 2 points (unable to place limb) [13]. The rats with a score of 8 indicated the most severe behavioral deficits after ICH; conversely, the rats with a score of 0 were considered normal.

### 4.9. Statistical Analysis

Results are expressed as the mean ± standard deviation of the mean based on the statistics. Statistical significance was determined using a one-way ANOVA or ANCOVA followed by the post hoc Tukey’s multiple comparison analysis test when necessary. All analyses were performed using IBM SPSS Statistics 25 (SPSS Inc., Chicago, IL, USA) and GraphPad Prism version 7 (GraphPad Software, San Diego, CA, USA). Results with a probability lower than 0.05 were considered statistically significant.

## 5. Conclusions

In conclusion, we have developed a targeted delivery system using FION nanoparticles tagged to stem cells and a magnet-embedded helmet. The delivery of stem cells resulted in an attenuated inflammatory response in ICH. The injection of SNMs provides protective effects and minimizes the potential side effects. Thus, the targeted delivery of SNMs might have the potential for emerging therapeutic strategies for ICH.

## 6. Patents

Patent number: *KR/10-2017-0092408*.

## Abbreviations

ESC	Embryonic Stem Cells
SNM	Spherical Neural Masses
ICH	Intracerebral Hemorrhage
PBS	Phosphate-Buffered Saline
FION	Magnetosome-like Ferrimagnetic Iron Oxide Nanocubes
NP	Neural Precursors
NSC	Neural Stem Cells
CCK-8	Cell Counting Kit-8
RITC	Rhodamine B Isothiocyanate
MPO	Myeloperoxidase
Ox-6	MHC Class II RT1B
IL-1β	Interleukin-1 Beta
ERK	Extracellular Signal-regulated Kinase
p-ERK	Phosphorylated Extracellular Signal-regulated Kinase
COX-2	Cyclooxygenase-2
MSC	Mesenchymal Stem Cells
SPION	Superparamagnetic Iron Oxide Nanoparticles
EB	Embryoid Bodies
DAPI	4,6-diamidino-2-phenylindole
PFA	Paraformaldehyde
BSA	Bovine Serum Albumin
